# Neural, biomechanical, and physiological factors involved in sex-related differences in the maximal rate of isometric torque development

**DOI:** 10.1007/s00421-016-3495-7

**Published:** 2016-11-04

**Authors:** J. Greig Inglis, Kyle McIntosh, David A. Gabriel

**Affiliations:** Electromyographic Kinesiology Laboratory, Faculty of Applied Health Sciences, Brock University, 1812 Sir Isaac Brock Way, St. Catharines, ON L2S 3A1 Canada

**Keywords:** Electromechanical delay, Muscle activation rate, Neural control, Strength testing, Surface electromyography, Muscle fiber conduction velocity, Contraction time

## Abstract

**Objective:**

Recent research has reported that lower maximal rate of torque development (dτ/d*t*_max_) exhibited by females, relative to males, during knee extension can be accounted for by normalization to a maximal voluntary contraction (MVC); however, this was not seen in the upper limb.

**Purpose:**

The aim of the current work was to examine the contribution of maximum strength (τ_max_), twitch contraction time (CT), muscle fiber condition velocity (MFCV), and rate of muscle activation (Q_30_) to sex-differences in the dτ/d*t*_max_ during maximal isometric dorsiflexion.

**Methods:**

Thirty-eight participants (20 males; 18 females) performed both maximal voluntary and evoked isometric contractions of the tibialis anterior across 3 days. Ten maximal compound muscle action potentials were elicited and subsequently followed by three, 5-s contractions. From the recordings, MFCV, dτ/d*t*_max_, τ_max_, CT, electromechanical delay (EMD), root-mean squared (RMS) amplitude, peak-to-peak voltage (Vpp), and Q_30_ were calculated.

**Results:**

An ANCOVA showed that τ_max_ accounted for all the sex-differences in dτ/d*t*_max_ (*p* = 0.96). There were no significant differences between groups with respect to MFCV, RMS amplitude, Vpp amplitude, or CT. However, there was a significant sex-difference in dτ/d*t*_max_, τ_max_, and Q_30_. Females had longer evoked EMD times compared with males (15.69 ± 10.57 ms versus 9.95 ± 3.46 ms; *p* = 0.01), but the voluntary EMD times were not different.

**Conclusion:**

The current research supports the work by Hannah et al. Exp Physiol 97:618–629, (2012) that normalization to MVC in the quadriceps is able to account for all sex-differences in rate of toque development in the lower limb.

## Introduction

The rate of tension development has received increased attention as a critical aspect of dynamic muscle performance during activities of daily living (e.g., balance maintenance) and sport performance (Aagaard et al. 2002; LaRoche et al. 2010; Paasuke et al. 2001; Pijnapples et al. 2008; Schultz et al. 1997; Tillin et al. 2013). A number of studies have shown that there is an inextricable link between the ultimate strength of the muscle and its rate of tension development (Andersen and Aagaard 2006; Holtermann et al. 2007). However, neural factors can also play an important role, as training-related increases in the maximal rate of tension development are associated with an increase in muscle activation at the onset of contraction (Van Cutsem et al. 1998; Van Cutsem and Duchateau 2005). Inglis et al. (2013) recently showed that neural factors also play a role in sex-differences in the maximal rate of tension development in the upper limb. Maximum strength was used as a covariate and was only able to account for a portion of the sex-differences. The addition of a second variable, the maximum rate of electromyographic (EMG) activity at the onset of contraction (Q_30_), was able to eliminate statistically significant sex-differences.

Given the inherent relationship between muscle strength and the maximum rate of tension development, few studies have explored additional mechanisms that would explain sex-differences in the maximal rate of tension development (Aagaard et al. 2002; Andersen and Aagaard 2006; Folland et al. 2014; Van Cutsem et al. 1998). However, sex-differences in lower extremity musculoskeletal injury rates (DiStefano et al. 2015) and falls incidences (Hess and Woollacott 2005; Stevens and Sogolow 2005) may be linked back to this critical aspect of muscle contraction (Bento et al. 2010). Hannah et al. (2012) explored potential neural and biomechanical factors involved in sex-differences in the maximal rate of tension development in the quadriceps, in addition to maximum strength. Twitch properties, electromechanical delay, and muscle activation using surface EMG (sEMG) were also assessed. Consistent with the more general findings for the relationship between maximum strength and the rate of tension development, when the peak rate of tension development was normalized with respect to maximum isometric strength of the muscle, the sex-differences were completely eliminated.

The results by Hannah et al. (2012) in the lower limb are contrary to the finding of Inglis et al. (2013) who showed a role for neural factors in the upper limb. It may be hypothesized that since males and females were more comparable in absolute strength in the quadriceps (Δ33%) than the biceps (Δ55.5%; Inglis et al. 2013), strength may entirely explain sex-differences in the rate of tension development in the lower limb. In contrast, the difference in maximum strength between males and females in the upper limb observed by Inglis et al. (2013) was much greater (Δ55.5%), possibly allowing for additional factors, such as the rate of muscle activation, to play a role. Inglis et al. (2013) explored the possibility that the interpretation of the results may differ based on normalization to maximum voluntary strength versus the use of a covariate approach. It was found that normalization to maximum voluntary strength also failed to account for all sex-differences in the maximal rate of tension development.

Since there is a strong relationship between maximum strength and the rate of tension development, sex-differences may be accounted for by maximum strength only when the two groups are ‘more’ comparable with respect to maximal strength as exists in the lower limb compared with the upper limb. For example, it has been shown that males and females are more comparable in maximal isometric dorsiflexion strength (Δ28.9%) and identical with respect to root-mean-square (RMS) sEMG magnitude (Heyward et al. 1986; Hoffman et al. 1979; Lenhardt et al. 2009). Unfortunately, Lenhardt et al. 2009 did not assess the maximum rate of torque development. The 28.9% strength difference between the sexes is consistent with Holmbäck et al. (2003) who concluded that muscle cross-sectional area was the principal determinant of dorsiflexion strength. In general, the muscle cross-sectional area of the TA in males is only 20% larger than that for females (Holmbäck et al. 2003; Jaworowski et al. 2002).

The purpose of this paper was to determine if sex-differences in the maximum rate of isometric dorsiflexion torque development are determined by maximum isometric dorsiflexion torque alone or other factors as observed for the upper limb where the strength differences are more pronounced. Based on the work of Hannah et al. (2012), it was hypothesized that maximum isometric strength would account for sex-differences in the maximum rate of torque development in the TA, because males and females are more comparable in maximum isometric strength in the lower limb. Studying mechanisms behind sex-differences in distal muscles that are responsible for balance and explosive activity can guide specific training interventions or rehabilitation techniques.

## Methods

### Participants

A power analysis was performed prior to data collection based on research by Lenhardt et al. (2009), showing that a subject pool of 18 males and females was sufficient to show differences in the rate of muscle activation (Q_30_). However, to protect against subject drop out, 40 subjects (males and females) were recruited. Pre-tension on the load cell was observed in two female subjects, so their data was removed from the analysis. Thus, 38 healthy Brock University Kinesiology students (20 males and 18 females) were analyzed in this study. The participants were free of any orthopedic or neuromuscular disorders, right leg dominant, and provided written informed consent prior to study participation in accordance with the Brock University Research Ethics Board guidelines (REB-02-284). Each participant was familiarized with the Electromyographic Kinesiology Laboratory prior to the first testing session. Prior to testing, participants were asked about their history of physical activity and weight training (years of experience) as well as the duration (hours per week and per day) and the percentage of weight training focusing on the upper body or lower body.

### Experimental setup and scheduling

All testing was performed as the participant sat in a custom built testing chair designed to isolate the dorsiflexors during maximal isometric contractions. Participants sat with their hip and knee joints secured at 90° of flexion and the ankle joint secured at 110° of plantar flexion (Inglis et al. 2011). Slight plantar flexion was chosen as previous research has shown that a certain degree of plantar flexion produces a maximal torque and 110° may place the TA closer to optimal length for both maximal evoked and voluntary dorsiflexion torque production, which considers the lever arm length (Marsh et al. 1981). A load cell (JR3, Woodland, CA, USA) was secured under the foot plate of which the foot was restrained by a minimally padded steel bar located proximal to the metatarsals for all torque recordings (Christie et al. 2009). There were 3 days of testing to assess the reliability of the measures as participants can exhibit a learning effect during maximal strength assessment (Green et al. 2014). On each day, participants were asked to perform the same tasks. These tasks included both voluntary isometric maximal dorsiflexion contractions and maximal evoked isometric torque. Each of the three testing days was separated by at least 48 h to avoid any complications, which may arise as a result of fatigue.

### sEMG recordings

Participants lay supine on a gurney, so that the most prominent TA motor point may be electrically identified using a metallic probe over the skin surface (Christie et al. 2005). The lowest possible current that produced a minimally visible twitch was taken as the motor point (Calder and Gabriel 2007). Following motor point identification, the recording areas were shaved, mildly abraded (NuPrep; Weaver and Co., Aurora, CO), and finally cleansed with alcohol to minimize skin–electrode input impedance. The sEMG recording electrode had three parallel stainless steel bars which resulted in two bipolar signals. Each stainless steel bar was 1 mm in diameter, 10 mm long, and was mounted with an interbar distance of 5 mm. The recording electrode was prepared with double-sided adhesive tape, electrolyte gel (Signal Gel; Parker Laboratories, Inc., Fairfield, New Jersey), and placed in line with the muscle fibers, 1 cm distal to the motor point. Alignment and final placement of the electrodes for recording MFCV followed the procedures outlined in McIntosh and Gabriel (2012).

Finally, a ground electrode (CF5000; Axelgaard) was placed on the lateral malleolus (McIntosh and Gabriel 2012). Electrode–skin input impedance (Grass EZM5, Astro-Med Inc., West Warwick, RI) was assessed before and after the experiment to ensure it remained below 10 kΩ. Skin temperature (Electrotherm TM99A; Cooper Instrument Corp., Middlefield, Connecticut) was also monitored before and after the experiment to verify that there was no change, which could affect the stability of the myoelectric signal.

sEMG was band-pass filtered (between 10 and 1000 Hz) and amplified (Grass P511; Astro-Med) to maximize the resolution on a 16-bit analog-to-digital converter (MI PCI-6052E; National Instruments, Austin, TX). All signals were collected at 5000 Hz and acquired on a computer-based data acquisition system (DASYLab; DASYTEC National Instruments, Amherst, New Hampshire). The data were stored on a PC (Celeron; Dell, Round Rock, Texas) for offline analysis.

The data window for the sEMG analysis of the maximal voluntary contraction was 500 ms, terminating before the middle of the contraction (Inglis et al. 2013). The sEMG signals were up-sampled to 25 kHz prior to calculating the cross-correlation coefficient to increase the time resolution of the action potential propagation (Farina and Merletti 2004). Muscle fiber conduction velocity calculation was based on the time delay identified by the peak of the cross-correlation function and the known interbar distance of 5 mm. The root-mean-square (RMS) amplitude of sEMG activity was also calculated. The rate of muscle activation was calculated by first rectifying the sEMG data and then numerically integrating the first 30 ms starting from the sEMG onset (Q_30_) that represents the rate of increase in the sEMG over the first 30 ms of muscle activity (Gottlieb et al. 1989).

Electromechanical delay (EMD) comprises an important portion of the rate of tension development phase of the contraction (Gabriel et al. 2001), where changes in motor unit activity patterns have been demonstrated to play a critical role in the maximal rate of torque development (Van Cutsem et al. 1998). Electromechanical delay was determined from the time lag between the onset of dorsiflexion torque and sEMG. sEMG onset threshold was identified as the first point of the sEMG signal to rise above the 95% confidence interval for baseline noise and to remain above the 95% confidence interval for 20 ms (Di Fabio 1987). Visual inspection was utilized to ensure the accuracy of the established threshold’s ability to detect either torque or sEMG onset (Inglis et al. 2013).

### Evoked isometric compound muscle action potentials

Compound muscle action potentials were evoked through a cathode stimulating electrode placed over the fibular nerve along with an anode placed on the medial condyle of the fibula to evoke an isometric dorsiflexion twitch contraction. The evoked potentials were monitored on an oscilloscope (VC-6525; Hitachi) to ensure that a consistent maximal response had been achieved for ten stimulations. The peak-to-peak voltage (Vpp) was extracted from the CMAP, while contraction time (CT) was obtained from the torque-time curve. Evoked contraction time (CT) was calculated from the time difference between the CMAP onset to its time to peak tension (Dahmane et al. 2005). Contraction time is used in this paper to separate differences in the rate of torque development associated with voluntary control versus muscle fibers properties. Ultimately, it was important to determine if potential sex-difference in Q_30_ could be due to the initial differences in peripheral factors (MFCV, Vpp, or CT) or overall voluntary activation (RMS).

### Maximal voluntary isometric dorsiflexion contractions

**Fig. 1:**
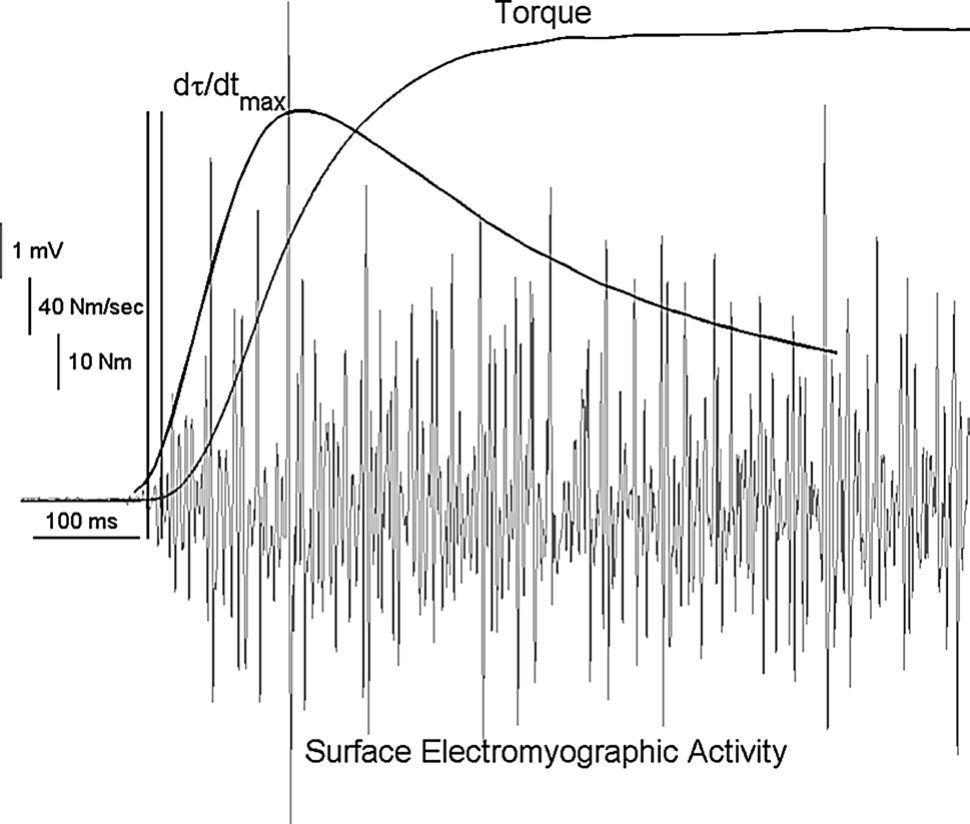
Torque (τ) (*dark grey*), surface electromyographic activity (*light grey*), and the rate of torque development (*black*) over the first second of a trial for a male representative subject. The *first vertical line* (*black*) represents the EMG onset; the *second vertical line* (*grey*) represents the torque onset

After the evoked contractions, a 15-min rest period was given. Participants then performed three voluntary maximal effort isometric dorsiflexion contractions by pulling with the top of their foot against a padded metal plate while minimizing toe extension. During the MVC’s, each participant was asked to contract “as hard and as fast as possible” with an emphasis on the “hard” (Sahaly et al. 2001). Each contraction lasted approximately 5 s in duration and was separated by a 5-min rest period. The force data were converted to torque values using the lever arm length, measured from the ankle joint to the metatarsals, where the load cell was located. A target line was given which represented 110% of the previously determined maximal effort, which was identified in real time on an oscilloscope. Furthermore, during each voluntary contraction, the participants were verbally encouraged to surpass the target line. Figure 1 shows a representative torque trace, rate of change in torque, and surface electromyographic activity of the tibialis anterior during a maximal effort dorsiflexion contraction. The manner in which the signals were collected is described below.

Maximum rate of torque development was calculated from the equation provided by Andersen and Aagaard (2006). The calculation involves determining the slope (Δτ/Δ*t*) over non-overlapping, successive 20 ms intervals, starting from the onset of the torque-time curve. The onset was determined as the point in the signal where the rate of change surpassed 1% of the maximal rate of torque development (dτ/d*t*_max_). The dτ/d*t*_max_ was then the maximum slope, which is synonymous with the ‘peak’ of the dτ/d*t* curve (Gabriel et al. 2001).

### Statistical analysis

Statistical analysis was conducted in two stages: first, to evaluate the reliability of the criterion measures using the intraclass correlational analysis of variance technique for males and females, separately; second, significant differences between males and females (Sex) in the magnitude of the means, and changes in the means across test sessions (Days), and the interaction (Sex × Days) was evaluated using a repeated-measures analysis of covariance to determine the impact of potential variables that may underlie sex-differences in the rate of torque development.

#### Intraclass correlation

Intraclass correlational analysis of variance (ANOVA) was performed to evaluate the reliability of the criterion measures for each group, which requires the consideration of both the stability of means and the consistency of scores. A one-way repeated-measures ANOVA was used to assess the stability of means across the three test sessions, while the intraclass correlation coefficient (model 2,k) was used to evaluate the consistency of scores within subjects. We adopted the convention delineated by Fleiss (1986) where an intraclass correlation coefficient (R) below 0.40 indicates poor reliability, between 0.40 and 0.75 is fair reliability, while values greater than 0.75 represent excellent reliability.

The magnitude of the intraclass correlation coefficient was further evaluated using the standard error of measurement (SEM) within an individual (Green et al. 2015). The SEM was calculated as the square of the mean square error for the ANOVA table using the variables ‘Sex’ and ‘Day’ (Weir and Cockerham 1984). The intrasubject coefficient of variation was the grand mean across the three test sessions divided by the SEM.

#### Analysis of covariance (ANCOVA)

A repeated-measures analysis of covariance (ANCOVA) was then used to determine the impact of a covariate on significant differences between males and females with respect to the dτ/d*t*_max_ development. Maximal torque and Q_30_ were the primary variables of interest. However, EMD, MFCV, and twitch contraction time (CT) were also explored. All statistical procedures were performed using SAS statistical software (SAS Institute Inc., Cary, NC) with alpha set at the 0.05 probability level. The means ± standard deviations (SD) for each measure are reported below.

## Results

### Participant characteristics

The means and standard deviations for the participant’s characteristics are given in Table 1. Significant differences were seen in all anthropometric measurements between males and females (*p* < 0.05). However, there were no significant differences in hours per week engaged in physical activity or weight lifting (*p* > 0.05). The grand means and standard deviations for the criterion measures across the three test sessions for males and females are presented in tables for the voluntary and evoked contractions (see Tables 2, 3).

**Table 1:** Demographic characteristics of the study participants

Measure	Females (*N* = 18) M ± SD	Males (*N* = 20) M ± SD	Difference%
Age (years)	24 ± 3.3	24 ± 2.4	0
Height (m)	1.6 ± 0.1	1.8 ± 0.1	11.1*
Mass (kg)	56.5 ± 8.5	79.7 ± 3.9	29.1*
Body mass index (kg/m^2^)	21.4 ± 2.2	24.4 ± 0.8	12.3*
Foot length (cm)	23.7 ± 0.7	28.5 ± 1.5	16.8*
Leg length (cm)	39.2 ± 1.8	47.3 ± 2.3	17.1*
Leg girth (cm)	37.0 ± 1.7	40.4 ± 1.9	8.4*
Physical activity (hours/week)	7.5 ± 3.4	9.3 ± 2.2	19.4
Weight-lifting (hours/week)	4.4 ± 3.7	5.5 ± 2.8	20.0

Significant differences were set at a *p* < 0.05 level and are indicated with *

**Table 2:** Data under the voluntary condition

**Female**	vMFCV (m/s)	vEMD (ms)	vRTDpk (Nm/s)	vTorque (Nm)	Q30 (mV x s)	RMS (mV)
Test Day	(M ± SD)	(M ± SD)	(M ± SD)	(M ± SD)	(M ± SD)	(M ± SD)
1	5.16 ± 1.61	37.19 ± 11.06	81.15 ± 33.65	29.10 ± 7.96	7.90 ± 5.27	0.21 ± 0.10
2	5.28 ± 1.40	33.86 ± 10.07	82.74 ± 36.02	29.17 ± 7.78	8.26 ± 6.97	0.21 ± 0.13
3	5.33 ± 1.69	27.09 ± 5.30	91.43 ± 34.88	27.10 ± 6.61	8.20 ± 6.74	0.17 ± 0.11
Grand	5.26 ± 1.55	32.71 ± 9.94*	85.11 ± 34.51	28.46 ± 7.39	8.12 ± 6.26	0.20 ± 0.11
*SEM*	0.67	8.02	10.35	2.76	3.06	0.03
*R*	0.81	0.35	0.91	0.86	0.76	0.92
**Male**						
Test Day						
1	4.92 ± 0.99	32.92 ± 12.48	160.03 ± 70.29	57.31 ± 17.12	5.80 ± 2.91	0.18 ± 0.11
2	5.13 ± 1.25	32.52 ± 12.08	157.04 ± 70.00	58.52 ± 21.69	4.83 ± 3.21	0.18 ± 0.13
3	4.70 ± 1.09	33.08 ± 13.74	142.01 ± 82.01	56.97 ± 20.54	5.26 ± 2.73	0.17 ± 0.08
Grand	4.92 ± 1.11	32.84 ± 12.57	153.03 ± 73.47	57.60 ± 19.55	5.30 ± 3.01	0.18 ± 0.11
*SEM*	0.55	6.12	30.29	8.06	1.88	0.06
*R*	0.76	0.70	0.83	0.83	0.61	0.72
*Percent Difference*	6.5	0.4	44.4*	50.6*	34.7*	10

Means (M) and standard deviations (SD) for voluntary muscle fiber conduction velocity (vMFCV), voluntary electromechanical delay (vEMD), voluntary peak rate of torque development (vRTDpk), voluntary maximum torque (vTorque), voluntary rate of EMG increase over the first 30 ms (Q_30_), and voluntary root-mean squared amplitude (RMS). Significant differences were set at a p<0.05 level and are indicated with *

**Table 3:** Data under the evoked condition

**Female**	eMFCV (m/s)	eEMD (ms)	eRTDpk (Nm/s)	eTorque (Nm)	Vpp (mV)	CT (ms)
Test Day	(M ± SD)	(M ± SD)	(M ± SD)	(M ± SD)	(M ± SD)	(M ± SD)
1	4.22 ± 1.76	16.71 ± 11.21	46.07 ± 18.41	2.59 ± 1.43	2.17 ± 0.92	77.20 ± 17.63
2	4.11 ± 0.99	15.38 ± 10.98	46.75 ± 18.43	2.69 ± 1.28	2.22 ± 0.91	80.74 ± 12.18
3	4.34 ± 1.51	14.97 ± 10.02	46.25 ± 14.43	2.68 ± 1.10	2.07 ± 0.98	69.21 ± 22.78
Grand	4.22 ± 1.43	15.69 ± 10.57	46.36 ± 16.87	2.66 ± 1.25	2.16 ± 0.92	75.72 ± 18.37
*SEM*	0.50	4.25	4.13	0.28	0.36	4.41
*R*	0.88	0.84	0.94	0.95	0.85	0.91
**Male**						
Test Day						
1	4.33 ± 1.50	10.57 ± 3.30	106.48 ± 39.66	5.89 ± 2.33	2.59 ± 1.14	68.65 ± 7.47
2	4.71 ± 1.66	10.17 ± 4.56	101.01 ± 41.67	5.54 ± 2.48	2.51 ± 1.13	79.55 ± 11.98
3	4.17 ± 0.99	9.12 ± 2.06	104.50 ± 35.12	5.86 ± 1.99	2.73 ± 1.01	76.60 ± 8.09
Grand	4.40 ± 1.40	9.95 ± 3.46	104.00 ± 38.31	5.77 ± 2.24	2.61 ± 1.08	74.93 ± 10.33
*SEM*	0.76	2.27	8.57	0.45	0.40	2.32
*R*	0.71	0.57	0.95	0.96	0.87	0.64
*Percent Differences*	4.1	36.6*	55.4*	53.9*	17.2	0.01

Means (M) and standard deviations (SD) for evoked muscle fiber conduction velocity (eMFCV), evoked electromechanical delay (eEMD), evoked peak rate of torque development (eRTDpk), evoked maximum torque (eTorque), evoked peak-peak voltage (Vpp), and the evoked contraction time (CT). Significant differences were set at a p<0.05 level and are indicated with *

### Reliability analysis

Table 2 shows that the consistency of scores within subjects for the criterion measures obtained during the voluntary contractions for females was excellent (*R* = 0.76–92) except for EMD which had an intraclass correlation coefficient of *R* = 0.35 (see Table 2). Female participants exhibited a 27.2% reduction in EMD from session 1 to session 3 (*F* [2, 51] = 5.83, *p* = 0.0045). The lack of stability, as assess by the repeated-measures ANOVA, resulted in a reduced intraclass correlation coefficient. However, the intrasubject coefficient of variation (Grand Mean/SEM) was 24.5%, which was deemed acceptable for further analyses (Green et al. 2015).

The means of the criterion measures generated during voluntary contractions as shown in Table 2 were highly stable across test sessions in males, while the consistency of scores within subjects ranged from fair to excellent (*R* = 0.61–83). The higher intrasubject variation (56.8%) for males Q_30_ resulted in a lower intraclass correlation coefficient (*R* = 0.61), but was still acceptable.

The same was true for the criterion measures generated during evoked contractions (*R* = 0.85–95) and Table 3 shows that consistency of scores within subjects for the criterion measures obtained during the evoked contractions in males ranged from fair to excellent (*R* = 0.57–96). The lowest intraclass correlation coefficient was for EMD, but it had an intrasubject coefficient of variation of only 22.8%. While there was a slight decrease in the means across sessions, a limited range of scores contributed to a decreased intraclass correlation coefficient. Thus, the measure was still deemed acceptable for further analyses.

### Between groups analyses

A repeated-measures ANOVA revealed a significant difference between groups and across days for voluntary maximal dorsiflexion isometric torque (*F* [1, 36] = 45.97, *p* = 0.001). Males had on average a 50.6% greater torque output than females. The difference between males and females with respect to the maximum rate of torque development was similar in magnitude. The maximum rate of torque development was 44.6% greater in males than for females (*F* [1, 36] = 16.46, *p* = 0.0003). In contrast, females had a 34.7% greater rate of increase in muscle activation as assessed by Q_30_ (*F* [1, 36] = 4.30, *p* = 0.0454). There were no significant differences between males and females with respect to EMD, RMS amplitude, or MFCV (*F* [1, 36] = 0.00, *p* = 0.9635; *F* [1, 36] = 0.31, *p* = 0.5834; *F* [1, 36] = 0.86, *p* = 0.3598, respectively).

Similar to the voluntary contractions, maximal evoked dorsiflexion torque was 53.9% greater for males than for females (*F* [1, 36] = 28.52, *p* = 0.001), and males also had a comparably greater maximum rate of torque development of 55.4% (*F* [1, 36] = 13.99, *p* = 0.0006). The EMD was 36.6% shorter for males than for females during the evoked contractions (*F* [1, 36] = 7.02, *p* = 0.0119). In contrast, MFCV, evoked CT, and Vpp of the CMAP were not significantly different between groups (*F* [1, 36] = 0.22, *p* = 0.6451; *F* [1, 36] = 0.03, *p* = 0.8589; *F* [1, 36] = 2.42, *p* = 0.1288, respectively).

### Analysis of covariance

When maximal torque was used as the covariate in the repeated-measures ANCOVA for the maximum rate of torque development, the difference between means decreased to 1.2% (*F* [1, 36] = 0.01, *p* = 0.9264). The rate of increase in muscle activation (Q_30_) was not assessed as a covariate, because females were actually greater than males. The only other significant difference between males and females was EMD during evoked contractions. However, evoked EMD had little impact as a covariate. Sex-related differences in the maximum rate of torque development were reduced to 33.8%, which were still significant (*F* [1, 36] = 8.27, *p* = 0.0067).

## Discussion

The main finding of the current work was that maximum torque was able to account for almost all of the sex-related differences in the maximum rate of torque development, as the percent difference in least-square means was reduced to 1.2% when using maximum torque as a covariate. There also were no significant differences between males and females in sEMG RMS amplitude magnitude as observed by Lenhardt et al. (2009). The absence of sex-differences in the sEMG signal amplitude, Vpp, and CT may reflect a comparable number of hours per week of training for the two groups (Aagaard et al. 2002). Contraction time was used in the study to determine if differences in the maximal rate of torque development were associated with either descending voluntary control or peripheral factors associated with muscle composition (Close 1972). Based on the fact, no sex-related differences in the CT (0.01%) were found suggests that the two groups were also similar with respect to muscle fiber composition, which is consistent with other research (Hicks and McCartney 1996).

Based on the work of Lenhardt et al. (2009), it was expected that males and females would be more comparable in maximum strength in the lower limb than the upper limb, which is consistent with other studies on sex-differences in maximum strength (Heyward et al. 1986; Hoffman et al. 1979). Instead, a 50.6% difference was observed, which was the same order of magnitude as previously observed for the upper limb (Inglis et al. 2013). One reason may be related to differences in the cross-sectional area (CSA) of the TA in the present sample versus that of Lenhardt et al. (2009). Although CSA was not directly measured, the males and females in this study had a larger difference in lower leg girth (8.4%) than the 5.2% observed by Lenhardt et al. (2009), which may suggest a larger difference in TA CSA.

The large difference in maximum strength between males and females was associated with a comparably large difference in the maximum rate of isometric dorsiflexion torque development (44.6%). Greater co-activation of the antagonist muscle group in females could contribute to both lower maximum strength and rate of torque development. Macaluso et al. (2002) showed that females may use greater antagonist co-activation to stabilize the joint due to greater joint laxity, smaller agonist musculature, and potentially lower quality muscle mass (torque/CSA), as has been seen in older versus younger adults (Solomonow et al. 1988; Thelen et al. 1996). However, antagonist co-activation was not measured in this study as it is markedly lower during isometric contractions compared with dynamic contractions and particularly lower when the muscle is placed in a shortened position as in this study (Pasquet et al. 2006).

There were several novel findings in this study. First, there were no significant differences between males and females with respect to MFCV. This result may be due to comparable muscle fiber diameters between the sexes, which is a large determinant of MFCV (Lange et al. 2002; Lindstrom and Magnusson 1977; Merletti et al. 1995; Nishihara et al. 2005; Zwarts 1989). It has been shown that type I (slow twitch) fibers are situated predominately in the anterior portion of the TA (Henriksson-Larsén et al. 1983). Moreover, it is a general result that, while males have larger fiber diameters than females, the type I muscle fiber diameters in females are larger than their type II muscle fibers. The small interelectrode distance (5 mm) will record from a small pick-up volume that encapsulates these superficial type I fibers of the TA, which are comparable in muscle fiber diameter between the sexes.

A second novel finding is that there were pronounced sex-differences in Q_30_ that were not evident in either the voluntary (RMS) or evoked (Vpp) sEMG magnitude. Females were significantly greater than males with respect to the rate of muscle activation as assessed by Q_30_. The difference increased further (4%) with repeated testing. This change was also associated with a significant training-related reduction in voluntary EMD associated with repeated testing. This reduction in voluntary EMG but “not” evoked EMG, and highlights the change in neural control (Gabriel and Boucher 1998). We suggest that the greater Q_30_ and reduction in voluntary EMD for females were associated with a different motor unit activity pattern at the onset of muscle contraction. However, it is not possible to distinguish exact motor unit behavior that may be responsible for the sex-related differences in the rate of increase in surface EMG. Van Cutsem et al. (1998) established a link between the maximum rate of isometric dorsiflexion torque, the rate of increase in sEMG, and the incidence of doublets associated with progressive resistive training. Similarly, Gabriel et al. (2001)found that only three training sessions were sufficient to produce an increase in the maximum rate of isometric elbow extension torque development and mean spike frequency of the sEMG signal during the torque development phase of the contraction in female participants.

Bojsen-Moller et al. (2005) reported a positive correlation between the rate of torque development and tendon structure stiffness, indicating that 30% of the variation in torque development can be accounted for by the tendons mechanical properties. Winter and Brookes (1991) further hypothesized that joint laxity might also play a role in the tension development phase of the contraction as reflected in the EMD. We believe that the greater rate of increase in sEMG activation for females reflects a compensatory mechanism to transmit force to the tendon more effectively, as evident in the observed decrease in voluntary EMD (Kubo et al. 2003; Wilkerson and Mason 2000; Winter and Brookes 1991). In support of this idea, Rozzi et al. (1999)have shown that females exhibited greater integrated sEMG activity upon landing from a jump as a compensatory mechanism for greater knee joint laxity, which includes both the musculotendinous unit and ligamentous restraint.

## Limitations

sEMG can only provide an indirect measurement of differences and underlying changes in motor unit activity patterns. Similarly, the use of CT to look at fiber composition differences only allows the association of either similarities or differences. Although the possible influence of different muscle structures and sizes in males versus females was discussed in the paper, not directly measuring it with ultrasound is a limitation. It was also assumed that there were differences in TA tendon stiffness without having actually measured it. Moreover, caution must be applied when extrapolating the results to older adults. The use of a healthy college aged population may not account for differences between the sexes with aging, such as the loss of type II fibers, which may render the sexes more similar in the 6th and 7th decades.

## Conclusion

The results support the work of Hannah et al. (2012) as maximal strength of the TA as a covariate accounted for nearly all of the sex-differences in the maximum rate of torque development. This was true, even though the difference in maximal strength was quite pronounced (50.6%). Thus, the hypothesis that maximum strength accounts for sex-differences in the maximum rate of tension development in the lower limb due solely to the fact that males and females are more comparable in maximum strength than in the upper limb was not supported. Rather, the maximum strength accounted for sex-difference in the maximum rate of tension development despite a large discrepancy in strength between males and females. Furthermore, females had a greater rate of increase in sEMG activation, and exhibited a significant reduction in EMD with repeated testing, suggesting that they might utilize a different motor unit activity pattern at the onset of contraction. Future research may focus on training modalities and rehabilitation techniques that could optimize RTD rather than only focusing on maximum strength to assist an older population in balance maintenance and recovery from imbalance.

## Abbreviations

ANOVA: Analysis of variance
ANCOVA: Analysis of covariance
CMAP: Compound muscle action potential
CSA: Cross-sectional area
CT: Contraction time
dτ/d*t*_max_: Maximum rate of torque development
EMD: Electromechanical delay
MFCV: Muscle fiber conduction velocity
MVC: Maximal voluntary contraction
Q_30_: Rate of increase in the sEMG over the first 30 ms of muscle activity
R: Intraclass correlation coefficient
RMS: Root mean square
RTD: Rate of torque development
SEM: Standard error of measurement
sEMG: Surface electromyography
SD: Standard deviations
TA: Tibialis anterior
τ_max_: Maximum torque
Vpp: Peak-to-peak amplitude
